# Supplementary figures and images for: Compared with Cotrimoxazole Nitroxoline Seems to Be a Better Option for the Treatment and Prophylaxis of Urinary Tract Infections Caused by Multidrug-Resistant Uropathogens: An In Vitro Study
Source: Antibiotics (Basel). 2021 May 28;10(6):645. doi: 10.3390/antibiotics10060645 (PMC8230139; doi:10.3390/antibiotics10060645)

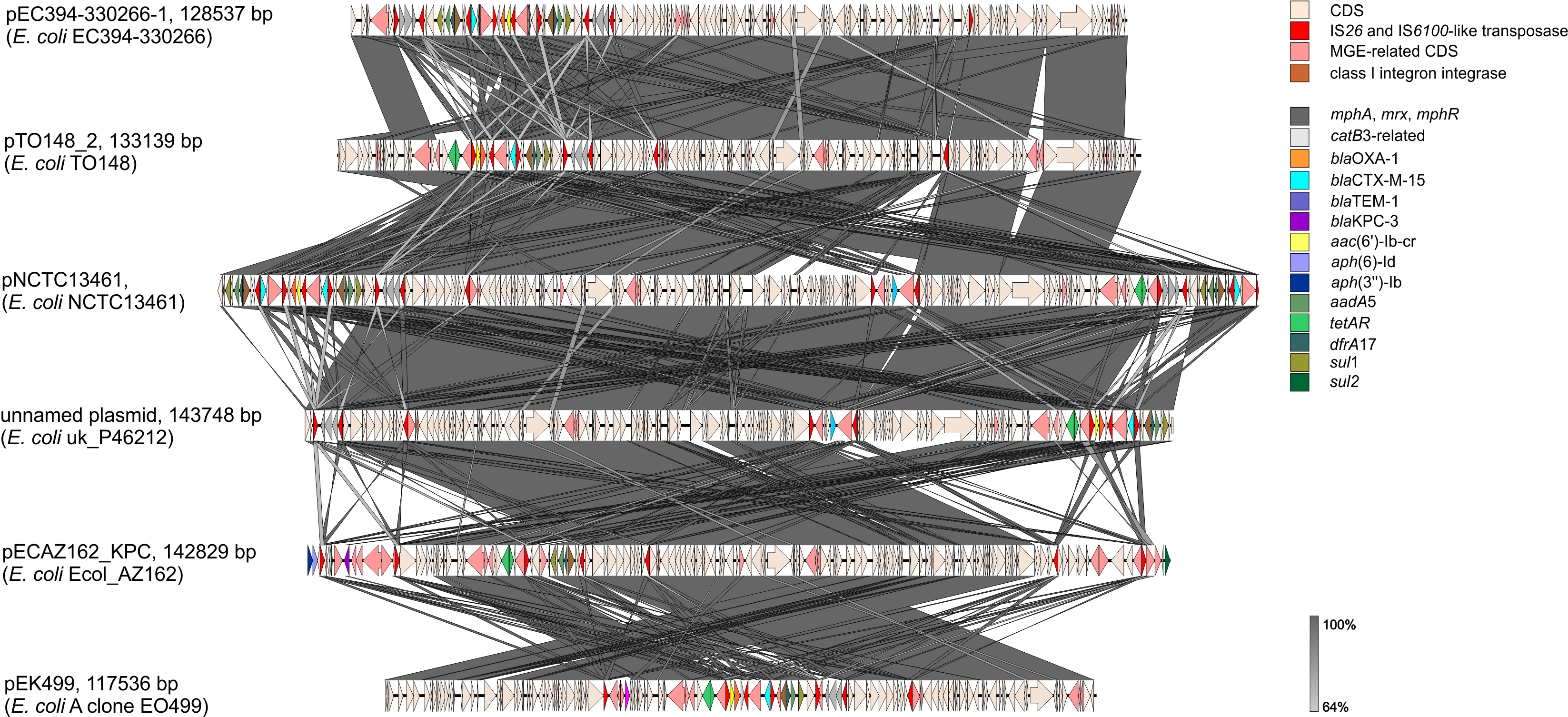

Supplement: Supplementary file 1 [file antibiotics-10-00645-s001.zip › FigS1_fin.jpg]
